# EasyMicroPlot: An Efficient and Convenient R Package in Microbiome Downstream Analysis and Visualization for Clinical Study

**DOI:** 10.3389/fgene.2021.803627

**Published:** 2022-01-04

**Authors:** Bingdong Liu, Liujing Huang, Zhihong Liu, Xiaohan Pan, Zongbing Cui, Jiyang Pan, Liwei Xie

**Affiliations:** ^1^ The First Affiliated Hospital of Jinan University, Guangzhou, China; ^2^ State Key Laboratory of Applied Microbiology Southern China, Guangdong Provincial Key Laboratory of Microbial Culture Collection and Application, Guangdong Open Laboratory of Applied Microbiology, Institute of Microbiology, Guangdong Academy of Sciences, Guangzhou, China; ^3^ Zhujiang Hospital, Southern Medical University, Guangzhou, China; ^4^ Department of Applied Biology and Chemical Technology, The Hong Kong Polytechnic University, Kowloon, Hong Kong SAR, China; ^5^ School of Public Health, Xinxiang Medical University, Xinxiang, China

**Keywords:** 16s rDNA sequencing, next-generation sequencing, microbiota, script, clinical data

## Abstract

Advances in next-generation sequencing (NGS) have revolutionized microbial studies in many fields, especially in clinical investigation. As the second human genome, microbiota has been recognized as a new approach and perspective to understand the biological and pathologic basis of various diseases. However, massive amounts of sequencing data remain a huge challenge to researchers, especially those who are unfamiliar with microbial data analysis. The mathematic algorithm and approaches introduced from another scientific field will bring a bewildering array of computational tools and acquire higher quality of script experience. Moreover, a large cohort research together with extensive meta-data including age, body mass index (BMI), gender, medical results, and others related to subjects also aggravate this situation. Thus, it is necessary to develop an efficient and convenient software for clinical microbiome data analysis. EasyMicroPlot (EMP) package aims to provide an easy-to-use microbial analysis tool based on R platform that accomplishes the core tasks of metagenomic downstream analysis, specially designed by incorporation of popular microbial analysis and visualization used in clinical microbial studies. To illustrate how EMP works, 694 bio-samples from Guangdong Gut Microbiome Project (GGMP) were selected and analyzed with EMP package. Our analysis demonstrated the influence of dietary style on gut microbiota and proved EMP package's powerful ability and excellent convenience to address problems for this field.

## Introduction

The in-depth understanding of human microbiome has dramatically reshaped our understanding of the relationship between human health and microbiome (Marchesi et al., 2016; Fan and Pedersen, 2021). A tremendous number of studies have demonstrated that microbiomes residing in the human body are key contributors in modulating host physiology and metabolism (Van Treuren and Dodd, 2020). As the second genome of the human being, the microbiomes are thought to be responsible for the complex pathophysiology nature of various diseases, e.g., neurological, metabolic, and immunity disorders (Oleskin and Shenderov, 2016; Cryan et al., 2019). Undeniably, the revolution in DNA sequencing technologies has enabled us to generate massive amounts of microbial data and accelerate the progression of studies and researches to explore the relationship between microbiomes and human health. Thus, a growing number of hospitals and medical centers endeavored largely to recruit volunteers and collect bio-samples associated with microbiomes (Claesson et al., 2017). For example, the Human Microbiome Project (HMP) in 2007 expanded our understanding of the microbiome across different body sites of a healthy person and its physiological roles in human genetic and metabolic landscapes (Peterson et al., 2009). Furthermore, emerging evidence indicate that microbiomes could be used as a non-invasive approach serving as novel diagnostic biomarkers and therapeutic targets. For example, 30 bacterial taxa identified from a cohort study could distinguish patients with early hepatocellular carcinoma with area under the curve (AUC) of 80.64% (Ren et al., 2018), and *Bacteroides vulgatus* may alter bile acid metabolism to improve the risk of polycystic ovary syndrome (Qi et al., 2019). In this regard, there is an urgent necessity to integrate microbial data into clinical practice for evidence-based medicine.

With the advancement of next-generation sequencing (NGS) and bioinformatics in basic and clinical biomedicine investigation, mathematics and statistical approaches in microbial downstream analysis are able to provide us comprehensive information of the relationship between microbiomes and human health and diseases (Knight et al., 2018). For example, diversity metric was introduced from ecology to access microbiota richness (Faith, 1992), while machine learning technology was popularly used for bacterial biomarkers screening (Vangay et al., 2019). In order to perform such measurements, clinical researchers usually have to take additional bioinformatics courses, which significantly obstruct the progression and frustrate amateurs without computational and coding experience (Knight et al., 2018). Here are three aspects of problems that clinical investigators face if they want to perform microbiome-related studies: First, clinical meta-data generally consist of a wide range of information including but not limited to age, body mass index (BMI), gender, and medical diagnostics, which brings about giant challenges for researchers to estimate and select proper features to determine inclusion criteria (He et al., 2018b). Moreover, in many retrospective studies, due to the complexity of subjects in hospitals, clinicians are not able to clearly determine grouping information based on meta-data, which challenges clinical researchers, especially various missing value in meta-data. Second, a large scale of microbial data always contains various information bias. For example, low abundance and occurrence taxa are often observed in microbial data analysis, which may be due to experimental contamination, sequence alignment error, and other factors. Normally, these taxa are filtered in downstream analysis according to study design and researchers' experience due to the lack of a well-recognized protocol, which may lead to biased and poorly reproducible results. Particularly, due to poor coding abilities, clinical researchers may find unexpected difficulties without enough knowledge in the data filtering step. Third, although many existing software (Caporaso et al., 2010) and R packages (Liu et al., 2021; Zhao et al., 2021) have been developed and integrated multiple methods from various fields, none of them are specially designed for clinical studies and could not address problems such as missing data, data filtering, and sample regrouping easily and efficiently. Moreover, due to large and comprehensive function and workflow, clinical researchers may spend additional time to learn and modify clinical data. The manual step to select the most appropriate parameters is still puzzling and tedious, and inconsistent application of such tools may reduce the reproducibility of the results. Thus, an efficient and convenient tool to meet the fast-developed clinical microbial studies is necessary.

Here, EasyMicroPlot (EMP) incorporates packages used in basic and clinical microbial studies for data analysis and visualization. In this package, regular downstream analysis covering core tasks of metagenomic analysis could be performed efficiently and conveniently in this field.

## Materials and Methods

### Package Description

**FIGURE 1:**
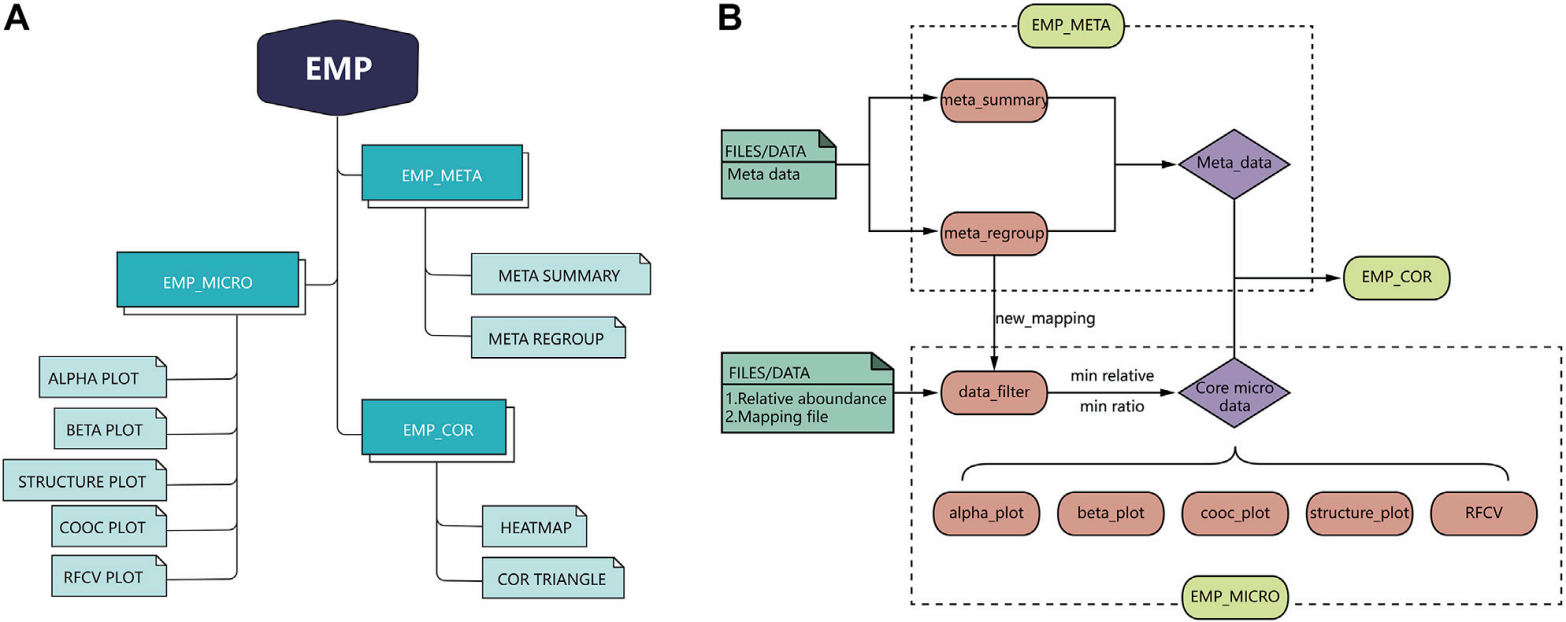
Overall design and workflow of EasyMicroPlot package.

EMP is developed based on R language 3.6 version and contains three main modules, which include EMP_META, EMP_MICRO, and EMP_COR. Compared to existing microbial analysis software in this filed, EMP extremely simplifies the whole process to the best and focuses on core microbiota and meta-data analysis in clinical studies. Each function in the EMP package is standalone and flexible, which enables users to design their own pipeline and utilize necessary functions without tedious parameterization and scripts. The overall design and workflow of EMP package is illustrated in Figure 1.

EMP_META module includes two functional units: the meta_summary and meta_regroup. The meta_summary function could enable users to easily visualize the distribution of missing value in meta-data, summarize basic information, and generate bivariate tables. The other function, meta_regroup, is designed to utilize various cluster analyses and 26 evaluation algorithms to determine the best regroup strategy based on different kinds of clinical information containing categorical and continuous variables.

EMP_MICRO module consists of data_filter, beta_plot, cooc_plot, structure_plot, tax_plot, RFCV, and RFCV_roc functions and mainly aims to provide investigators a fast and simple approach to accomplish the core tasks of data filter, such as *α*-diversity analysis, *β*-diversity analysis, co-occurrence network analysis, taxonomic stack bar plot, and random forest models for key taxa screening. The function EMP_MICRO could automatically identify data directly from R workspace and transform these into core microbial data at six levels (phylum, class, order, family, genus, and species). The feature allows users to activate a complete workflow with default parameters and generate results in workspace by applying function EMP_MICRO with only microbial abundance files and mapping file in user's R workspace. Most analysis functions including *α*-diversity, *β*-diversity, and taxonomy boxplot not only provide Student *t*-test and one-way analysis of variance (ANOVA) comparison methods but also offer an interactive plot in html format, which means users could easily identify outliers and recognize abnormal samples. Moreover, most of the existing microbial analysis software suggest investigators to provide well-matched microbial abundance files and mapping files. In this case, investigators have to modify all the files if they want to perform sub-group analysis or regroup analysis. To avoid such problem, EMP is designed in a way that users only need to edit their mapping file without modifying microbial abundance data.

EMP_COR module is designed to integrate metagenomic and clinical data. Investigators can explore links between meta-data and microbial abundance using Pearson index and Spearman and Kendall index, and we have developed two artistic styles for data visualization in this module.

### Data Preparation

To test our package, we selected a part of 16S rDNA sequencing data from Guangdong Gut Microbiome Project (GGMP) (He et al., 2018b). This dataset is composed of samples from a population in Shenzhen, China, of GGMP. A total of 618 16S rDNA sequencing data with meta-data including diets, districts, defecation, and metabolic syndrome (MetS) status in Shenzhen province was included in this analysis. Microbial relative abundance was generated at phylum, class, order, family, genus, and species levels using a standard QIIME 1.91 pipeline. All meta-data and microbial abundance were deposited in the Supplementary material.

## Results

### Subjects Enrollment

**FIGURE 2:**
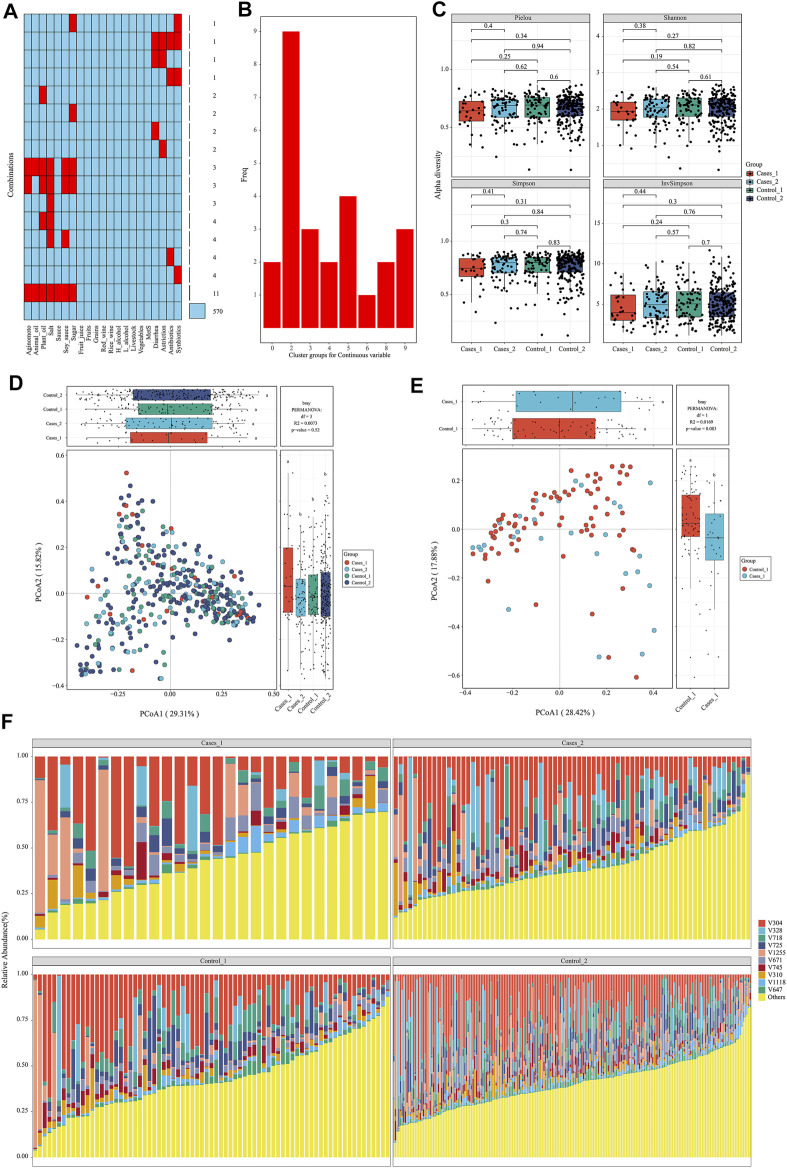
Diets are associated with significantly structural changes of gut microbiota. **(A)** The distribution of missing value in the meta data. **(B)** Twenty-six estimate indices vote for the best cluster number based on dietary structure. **(C)** *α*-Diversity on Pielou, Shannon, Simpson, and InvSimpson index among different subgroups. **(D, E)** *β*-Diversity on Bray–Curtis index and permutational MANOVA test among different subgroups with consideration of dietary structure. **(F)** The structure plot for top 10 gut bacterial taxa.

After data preparation, the function meta_summary in EMP_META could map the distribution of missing data and generate a general summary of meta-data based on MetS status (Figure 2A and Supplementary material). There are more than 20 missing information in features of “salt,” “plant oil,” “soy sauce,” and “sugar” intake. A three-line table also showed detailed dietary structure information among groups (Supplementary Table S1). Consider that gastrointestinal disorder, antibiotic therapy, and probiotics are closely linked to the dysbiosis of gut microbiota. Finally, only 394 samples were qualified and included into downstream analysis, and those who have experience of diarrhea, astriction, antibiotics, and synbiotics were excluded. In order to explore the microbial difference without bias of dietary pattern, the function of meta_regroup incorporated 26 indexes to estimate the cluster for dietary structure to determine the best regrouping design utilizing “Kmeans” and “Euclidean” parameter (Figure 2B). After calculation for continuous and categorical variables, 394 samples were included into downstream analysis and divided into four groups based on dietary structure and MetS status (Control_1: subjects without Mets whose dietary structure belong to type 1; Control_2: subjects without Mets whose dietary structure belong to type 2; Cases_1: subjects with MetS whose dietary structure belong to type 1; Cases_2: subjects with MetS whose dietary structure belong to type 2). Those who have experience of diarrhea, astriction, antibiotics, and synbiotics were excluded.

### Diets Are Associated With Significant Structural Changes of Gut Microbiota

To avoid interference of rare taxa, species data whose relative abundance was below 1‰ or prevalence rate was not more than 70% in any group was excluded using function of data_filter. Function structure_plot provided a general composition picture for these core data at species level (Figure 2F and Supplementary Material). With this core microbiota in hand, rarefaction measurement of Pielou, Shannon, Simpson, and InvSimpson index showed *α*-diversity difference was not significant with each other (*p* > 0.05) (Figure 2C). *β*-Diversity calculated with Bray–Curtis distance showed samples in Cases_1 group was far away from the other three groups in two-dimensional space (Figure 2D), which indicated these microbiota structures for MetS subjects with type A diet were significantly different from others (least significant difference *p* < 0.05). Particularly, when only two groups including Cases_1 and Control_2 were performed in PCoA analysis, permutational multivariate analysis of variance (MANOVA) test was almost statistically significant (*r* ^2^ = 0.01, *p* = 0.083) (Figure 2E). In contrast, we also performed *β*-diversity with the same parameter and could not observe significant change, which suggested diets indeed disturb the structure of the microbiota (Supplementary Figure S1).

### Diets Perturb the Ecology and Network of Gut Microbiota

**FIGURE 3:**
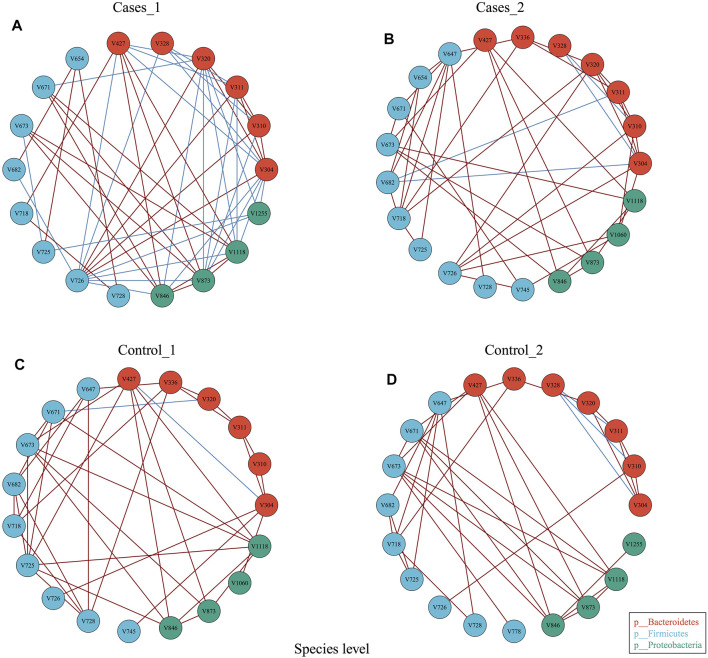
Co-occurrence analysis of bacterial interaction under different dietary pattern and MetS status.

In order to explore whether diet may influence the gut microbiota community network, EMP provides an easy function to perform co-occurrence analysis and generate network plot for each group. Co-occurrence analysis at species level with parameter of Spearman confident index [abs(r) > 0.3, *p* < 0.05] showed each group has almost the same vertices but presented totally different cross-talk among core gut bacterial taxa (Figure 3). For example, Control_1 group has unique V745 (*Veillonellaceae* *Phascolarctobacterium*) and V1060 (*Alcaligenaceae* *Sutterella*) in its network, while Cases_1 has V328 (*Prevotella copri*) and V1225 (*Aeromonadales* *Succinivibrionaceae*) in its network. Another comparison demonstrated that other than the common species between Control_2 and Cases_2, Control_2 group has V1225 (*Aeromonadales* *Succinivibrionaceae*), while case_2 includes additional three species, that is, V654 (*Clostridiaceae* *Clostridium*), V745 (*Veillonellaceae* *Phascolarctobacterium*), and V1060 (*Alcaligenaceae* *Sutterella*). Particularly, Cases_1 group with high network complexity (transitivity = 0.6315789, centralization degree = 0.3006536, graph density = 0.3464052) was obviously higher than others (Supplementary material), which suggested different dietary structures may change the systemic ecology of gut bacteria.

### Diets Significantly Interfere With the Accuracy of Random Forest Prediction for Patients With MetS

**FIGURE 4:**
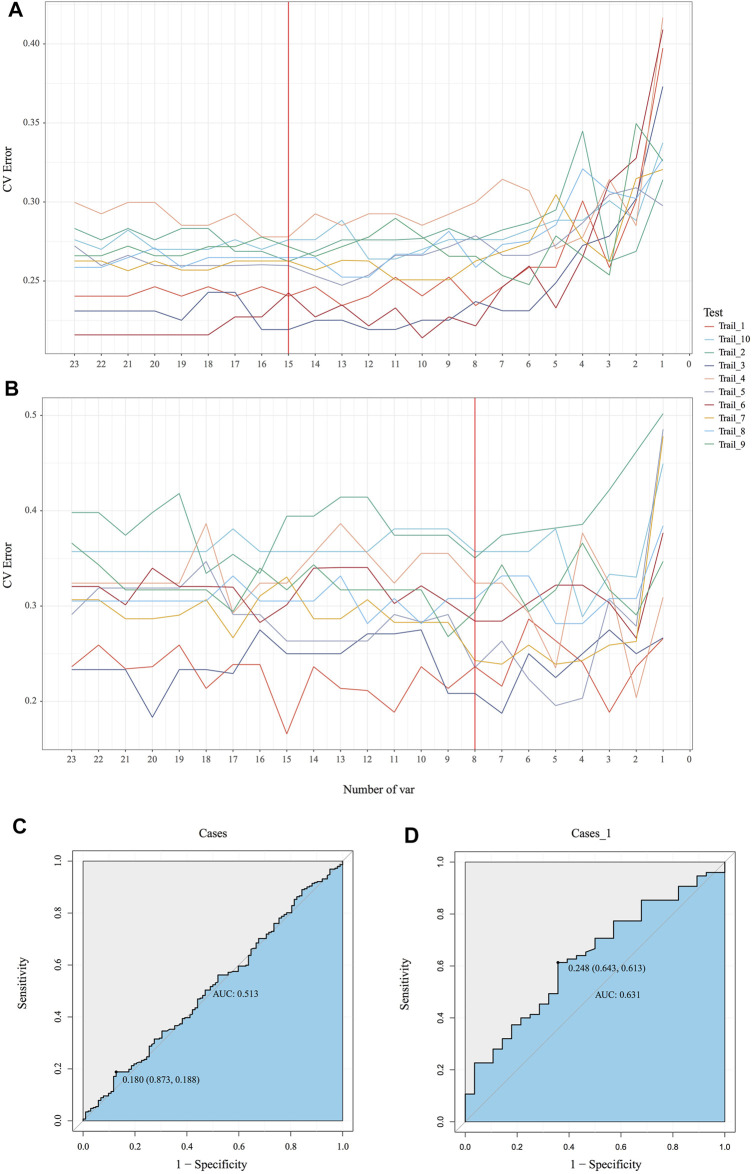
Identification of the signature gut microbiota by random forest. **(A, B)** To explore the signature biomarkers, a fivefold cross validation together with random forest was performed. **(C, D)** Based on key bacterial taxa generated by EMP package, receiver operating characteristic curves (ROC) were performed to test prediction models.

Emerging evidence proved microbiota could be characterized as markers for clinical auxiliary approach. As the most popular machine learning, random forest together with cross validation could robustly select key bacteria as biomarkers to build prediction model. In our MicroEasyPlot, RFCV function allows users to utilize relative abundance data to generate random forest prediction model and select potential maker taxa according to mean and standard deviation at a series of random number. With this, we constructed random forest model together with cross validation and explored microbial biomarkers to distinguish individuals with MetS from healthy ones (Figure 4B, Supplementary Figure S2, and Supplementary material). Fifteen bacterial taxa at species level were considered to be the most important biomarkers by a union of 10 random processes, while V647 (*Clostridia Clostridiales*), V671 (*Clostridiales* *Lachnospiraceae*), V725 (*Faecalibacterium prausnitzii*), V726 (*Ruminococcaceae* *Oscillospira*), and V718 (*Clostridiales* *Ruminococcaceae*) changed between groups significantly (*p* = 0.0088, 0.095, 0.018, 0.04, 0.088) (Supplementary Figure S3). RFCV_roc function also could be used to test this prediction model, through which we established receiver operating characteristic curve with AUC area 0.63 (Figure 4D). As a control, random forest model with the same parameters was performed to test the relative abundance data directly without subgroup analysis; AUC area only achieved 0.51, which indicated dietary style affected gut microbiota composition indeed and should be included into downstream microbial analysis in clinical studies (Figures 4A, C).

### Identification of the Relationship Between Dietary Structure and Microbial Abundance

**FIGURE 5:**
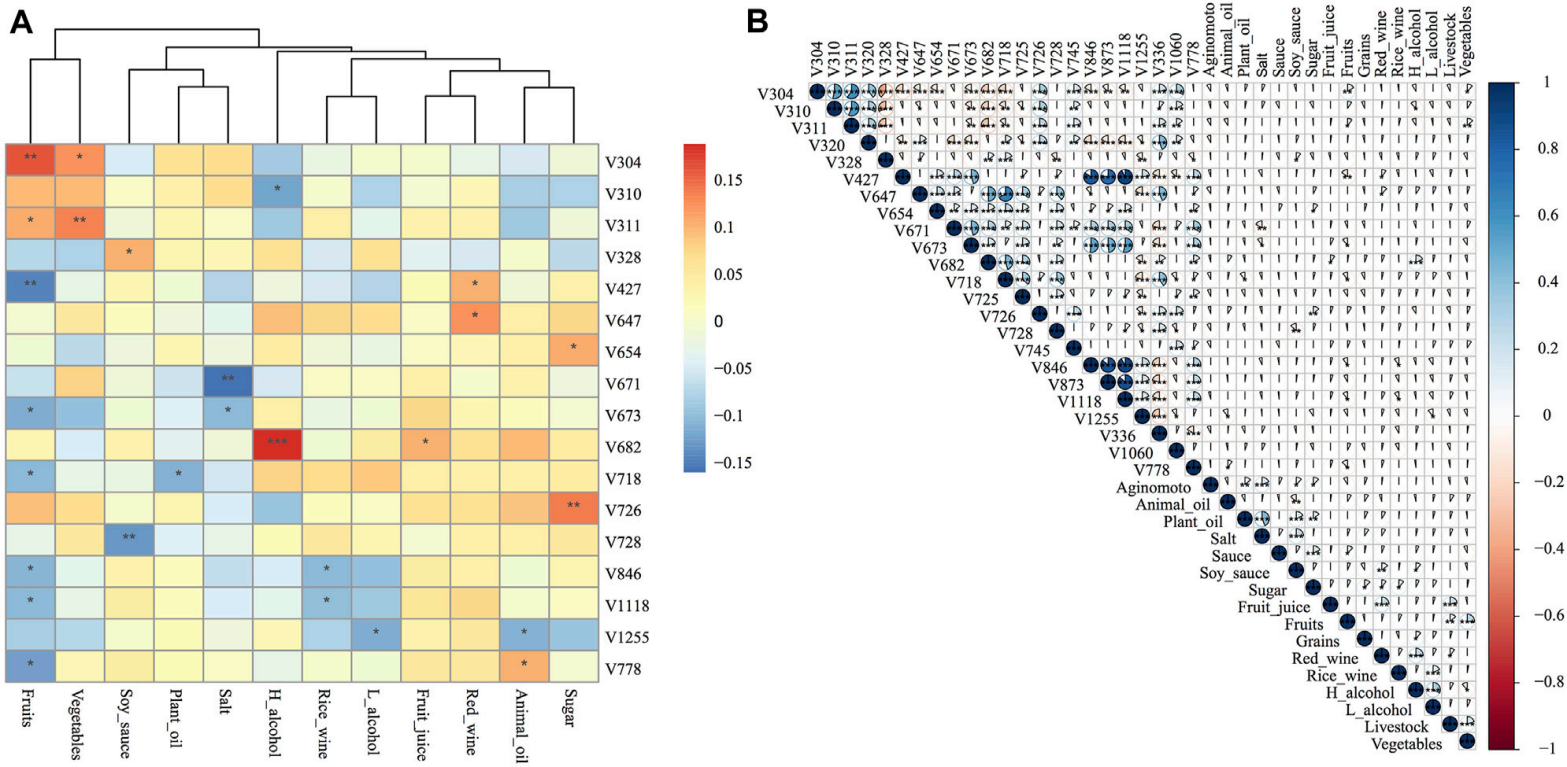
Correlation analysis between relative abundance of core bacterial taxa and meta data in clinical study.

To explore the detailed relationship of diets and core microbiota, function cor_plot_heat and cor_plot_detail module provides two kinds of visualization using “pearson,” “spearman,” and “kendall” measurement. Correlation analysis showed 17 species of 23 core taxa generated from data_fiter function were strongly associated with dietary changes. Especially for several key taxa identified by random forest model below, V647 (*Clostridia Clostridiales*) was positively correlated with red wine intake (*r* = 0.121, *p* = 0.017), V726 (Ruminococcaceae *Oscillospira*) was positively correlated with sugar (*r* = 0.136, *p* = 0.007), V671 (*Clostridiales* Lachnospiraceae) was highly correlated with salt consumption (*r* = −0.163, *p* = 0.001), and V718 (*Clostridiales* Ruminococcaceae) was highly correlated with fruits (*r* = −0.107, *p* = 0.034) and plant oil (*r* = −0.113, *p* = 0.025) (Figures 5A, B and Supplementary material). On the other hand, vegetarian diet including fruits, vegetables, and fruit juice influenced nine core gut bacterial taxa, which was considered to be the most influencing factor. High- and low-degree alcohol affected three taxa core gut bacteria, while red and rice wine disturbed four taxa. Seasoning including salt, sugar, soy sauce, and plant and animal oil also presented close relationships with various gut bacteria.

## Discussion

Due to the advent of bioinformatics and high-throughput sequencing technology, bioinformatics has become a well-qualified tool in establishing auxiliary diagnostic measurement in clinical practice (Kordahi et al., 2021). Notably, microbiome has gained more attention in fields investigating the biological and pathological nature of various diseases (Claesson et al., 2017). However, clinical researchers often encountered several difficulties in data analysis and visualization. In order to fill the gap between clinical researchers and microbiome data mining, we collected 16S rDNA sequencing data set from GGMP and performed data analysis with EMP to present the convenience and professional practice of our tool. With this dataset, we proved dietary pattern is an important contributor to different gut microbiota patterns.

Notably, huge meta-data analysis with detailed participants' information usually brings tremendous problems. Employing inappropriate strategy to estimate the features including continuous and categorical variables may lead to unexpected bias and errors. For example, dietary pattern could dramatically change or in the long term reshape the composition of gut microbiota (Singh et al., 2017). However, in previous studies, researchers may ether ignore the effects of diets in downstream microbial analysis or divide subjects into different groups according to dietary classification such as Western diet, Mediterranean diet, Vegetarian diet, etc. (Bian et al., 2017; Garcia-Mantrana et al., 2018). In the present study, 394 qualified subjects were selected from 618 volunteers in Shenzhen, Guangdong province of China. However, in the process of 26 estimating votes under different algorithms, “Kmeans” successfully divided them into two groups based on “Euclidean” distance, which indicated those 394 subjects from the same districts had two different dietary structures. Among the regrouped subjects only based on MetS status, the present study observed more changes of microbial structure and diversity under different dietary status indeed. Regrouping based on diet also improved the robustness of random forest prediction and increased AUC area in receiver operating characteristic curves (ROC) model. Additionally, correlation analysis further confirmed dietary components including fruits, vegetables, alcohol, sugar, oil, and salt significantly alter the core bacterial taxa. Animal studies have revealed that additional salt supplement could significantly deplete genera Clostridia, which was consistent with our observation (Wilck et al., 2017). Furthermore, in two large cohort studies (1,879 middle-aged elderly Chinese adults from Guangzhou Nutrition and Health Study and 6,626 subjects from GGMP), dietary fruit and vegetables were also proved to reshape gut microbiota (Jiang et al., 2020). Altogether, these results confirmed the importance of regrouping based on diet, suggesting microbiome-related clinical studies should take the dietary factor into consideration for participants' regrouping. Moreover, factors including smoking, education, life style, and others could also exert a great influence on the structure and diversity of gut microbiota (He et al., 2018a). A big cohort study demonstrated prolonged sedentary lifestyle may increase the prevalence of MetS through modulation of gut microbiota (He et al., 2018a). Other studies also suggested various sports and exercise could reshape human microbiota. Of note, elite athletes harbor several special taxa in the gut, which were proven to be able to catalyze lactate into propionate to extend running time (Scheiman et al., 2019). Thus, multiple factors should be taken into consideration and estimate their influence carefully in microbiota-related studies.

Secondly, low abundance and prevalent bacterial taxa may affect reliability and reproducibility of microbial-related studies and analysis and were thus considered to be contaminants (Claesson et al., 2017). Researchers have reached a consensus that it was hard for bacteria with low abundance to exert significant effects on the host, and taxa with low prevalence were likely to lead to false positive and negative results in classification and prediction models (Knight et al., 2018). For example, in our previous study, random forest model based on species data observed several markers in mathematics to distinguish patients with insomnia from healthy control, while many taxa determined by classification model without decontamination were actually outliers and lack biological significance between groups (Liu et al., 2019). Another cohort study has also set a strict decontamination standard with >0.5% of relative abundance and >30% of prevalence in downstream analysis to avoid potential bias (Biagi et al., 2016). Thus, an appropriate threshold for data filter is extremely necessary. Though the concept of filtering microbial data is well accepted in microbial studies, there is no professional tool in this area. Data analysts always filter data by self-developed script, while others even modified data in excel format manually. EMP package provides a convenient function, data_filter, to address this problem. Bacteria could be excluded by two thresholds including minimum abundance and prevalence, which means users could easily customize the filter according to study design and generate core bacterial taxa in one step. Before the application of data filter function, a total of 1,503 species annotated from 394 feces samples were generated, and a handful of taxa only presented in few samples with low abundance. In terms of biologic aspect, these taxa were believed to originate from contamination and annotation error and may have an adverse effect on downstream computation. Utilizing data filter function in EMP package with 0.001 minimum relative abundance and 0.7 minimum prevalence threshold, only 23 core species were qualified for the following analysis, which dramatically economized the computational resources and reduced the bias and errors. Among these core species, 17 taxa were proven to be highly correlated with diet, which further confirmed the value of data filter function. For the first time, clinical researchers could easily decontaminate microbial data sets and generate core bacteria for downstream analysis with a well-recognized process.

Third, solid and meaningful results were normally generated under standardized and scientific approaches in data analysis. Although tremendous tools and online platforms were developed in the past decade, clinical researchers without coding experience were not satisfied with the complicated instruction or limited functions. Particularly, certain tutorials in written books or online websites about microbial data analysis only offer sets of scripts containing the usage of many independent software, and following such tutorials is time consuming. Even worse, it is common to see codes shared by publishers containing kinds of errors without peer review, including but not limited to the inappropriate usage of certain software and tools. Additionally, most of the researchers did not provide detailed script pipeline, and editors merely require researchers to upload key codes and scripts in Supplementary material or open-source platform due to the complexity of script. Without confirmation of the correctness of the self-written codes, it is hard to realize the unexpected false positive and false negative conclusion, and this makes it impossible to reproduce the computational results. On the other hand, researchers also need an easy way to design their own pipeline to continue attempting and computing results many times. Given a lot of independent software were integrated into code text by hand, collaborators may find it different to read and use, which may largely increase the risk of error and bugs. Thus, after collecting and screening popular analysis strategy, EMP package divides the whole analyzing process into three modules, and each module could be utilized separately, which provides enormous convenience in research work. In the present study, EMP package helped to estimate missing data and classify 394 samples into four groups according to dietary structure. After receiving group information, Microplot module could simply analyze microbial data in one script covering *α*-diversity, *β*-diversity, co-occurrence, structure plot, and random forest models. At last, correlation analysis revealed the influence of dietary structure on gut microbiota.

There are three main advantages of the current EMP package: First of all, packages integrated into EMP package are well accepted by users in this field and documented on the Comprehensive R Archive Network (CRAN). All of these packages are widely utilized to perform microbial data analysis and visualization. Moreover, EMP package is an open-source tool, and users are welcome to report any bugs. Second, the existing tools and R packages made great effort to incorporate a wide range of microbial analysis approaches and statistics method, while EMP package focuses on clinical studies, and the whole process is divided into three parts for the core microbial data analysis. Given many retrospective studies cannot determine groups for samples, EMP provides scientific method to help clinical scientists screen and regroup samples. Besides, EMP package does not need well-matched relative abundance files and mapping file and could automatically identify bacterial level and perform data analysis according to mapping file containing samples identifiers and group information in text format or data frame generated from R script without modifying bacterial data, which may significantly reduce mistakes in many attempts. Third, in order to maximally simplify the operating procedure, EMP package allows users to perform the whole workflow with only one step and generate all results in the workspace. Each core analysis in workflow also could be performed by applying one function, which means researchers could design their own pipeline in a few lines of script with modules they are interested in for the study. In this case, EMP simplified clinical users' self-developed codes, allowing peer reviewers and readers to also test and reproduce specific results with few codes. Thus, with EMP package, clinical investigators could explore a huge scale of clinical data together with microbial abundance information and publish their result easily and reliably.

## Conclusion

EMP package incorporates widely used microbial data analysis and visualization tools deposited in CRAN and provides clinical investigators with a convenient approach to perform downstream data filtering, analysis, and visualization. From the demo data, we demonstrated that researchers could simply utilize different modules to identify missing data, classify patients into different groups, and regroup them based on different parameters. Most importantly, this package could help clinicians robustly select key microbial biomarkers and calculate the correlation index between core microbiota and clinical parameters, such as BMI, age, and height, etc. Overall, EMP package provides an efficient and convenient downstream microbiome analysis pipeline, especially for clinical investigators without additional script experience.

## Data Availability

The datasets presented in this study can be found in online repositories. The names of the repository/repositories and accession number(s) can be found below: https://github.com/xielab2017/EasyMicroPlot.
