# Supplementary figures and images for: EasyMicroPlot: An Efficient and Convenient R Package in Microbiome Downstream Analysis and Visualization for Clinical Study
Source: Front Genet. 2022 Jan 4;12:803627. doi: 10.3389/fgene.2021.803627 (PMC8764268; doi:10.3389/fgene.2021.803627)

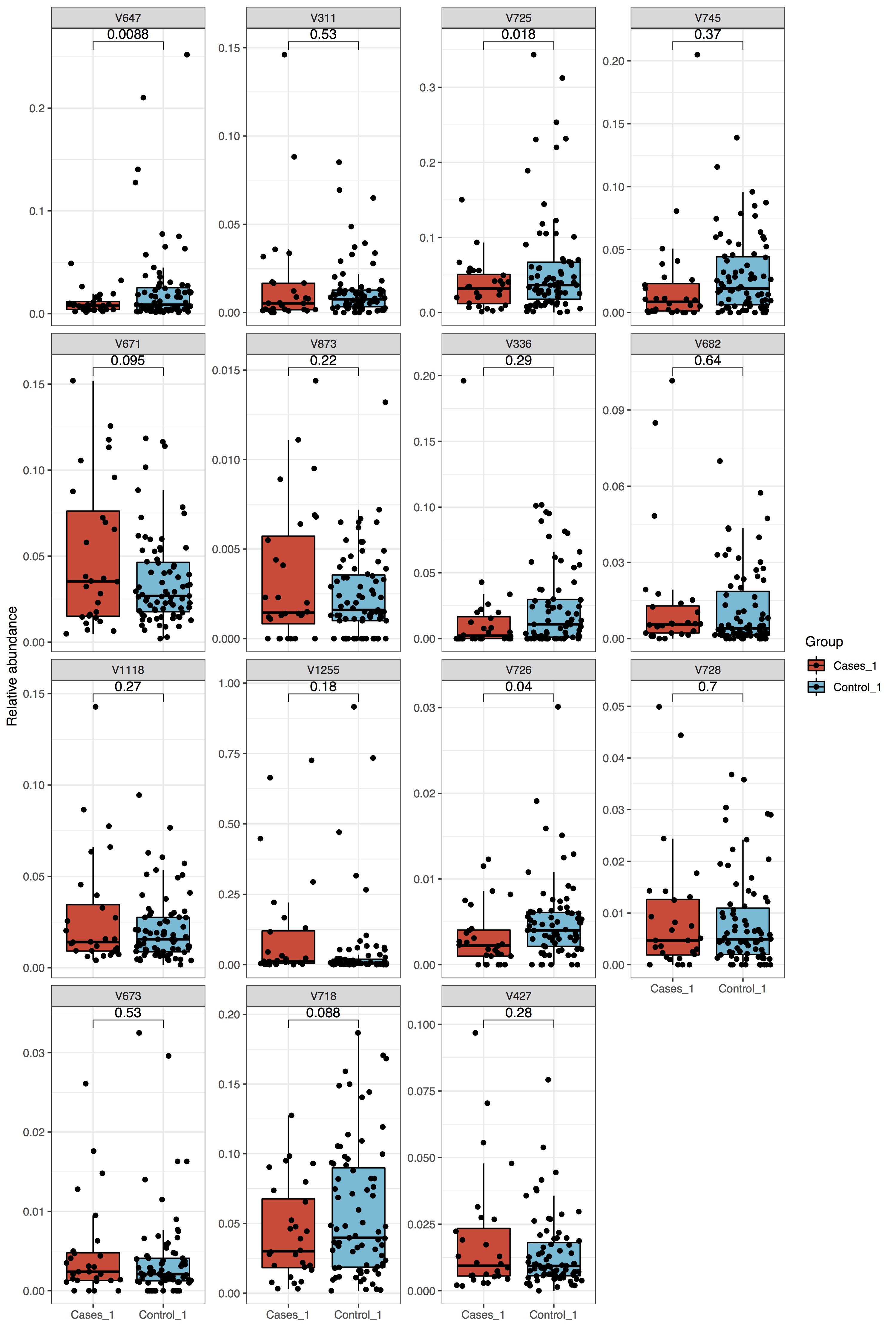

Supplement: Supplementary file 1 [file Image3.JPEG]

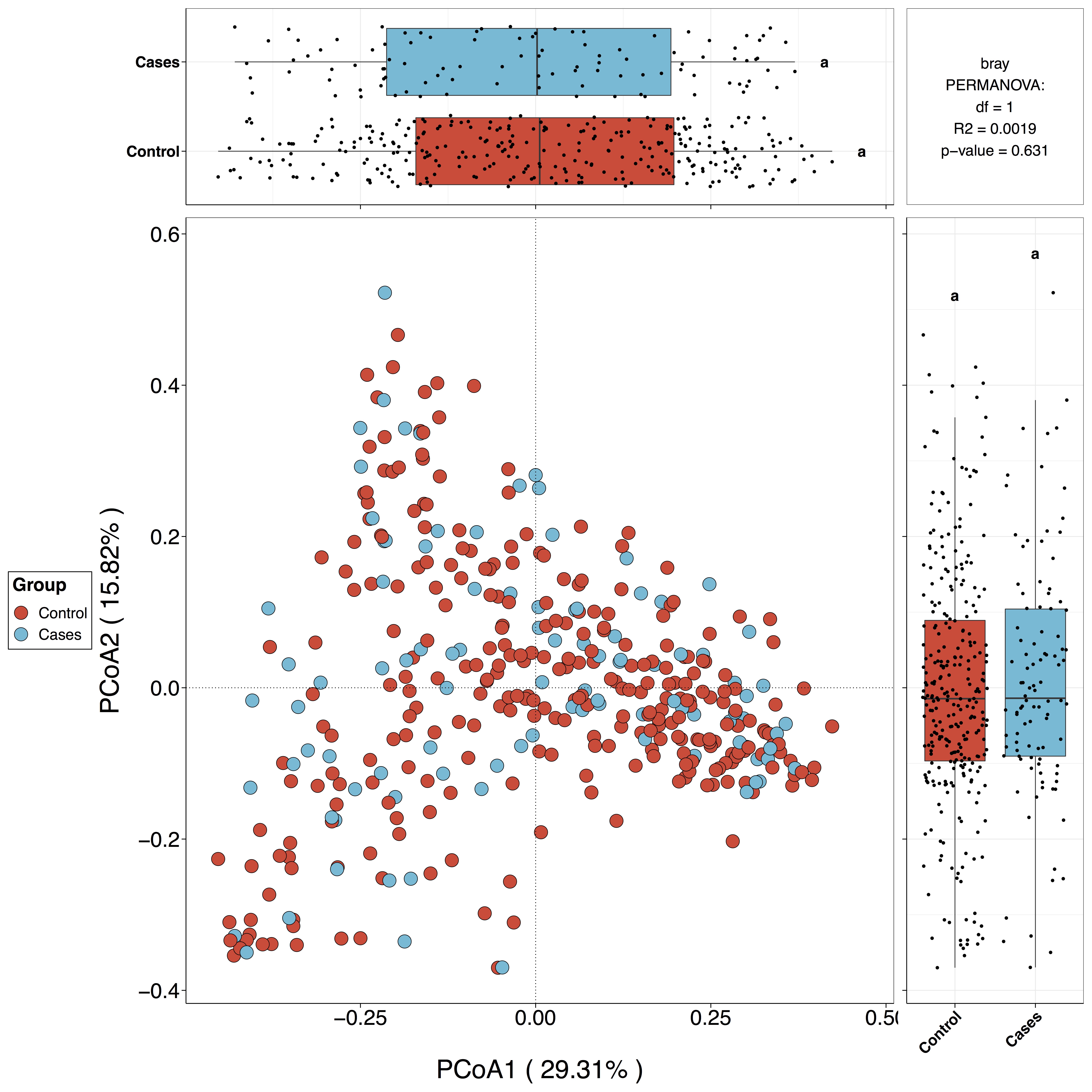

Supplement: Supplementary file 2 [file Image1.JPEG]

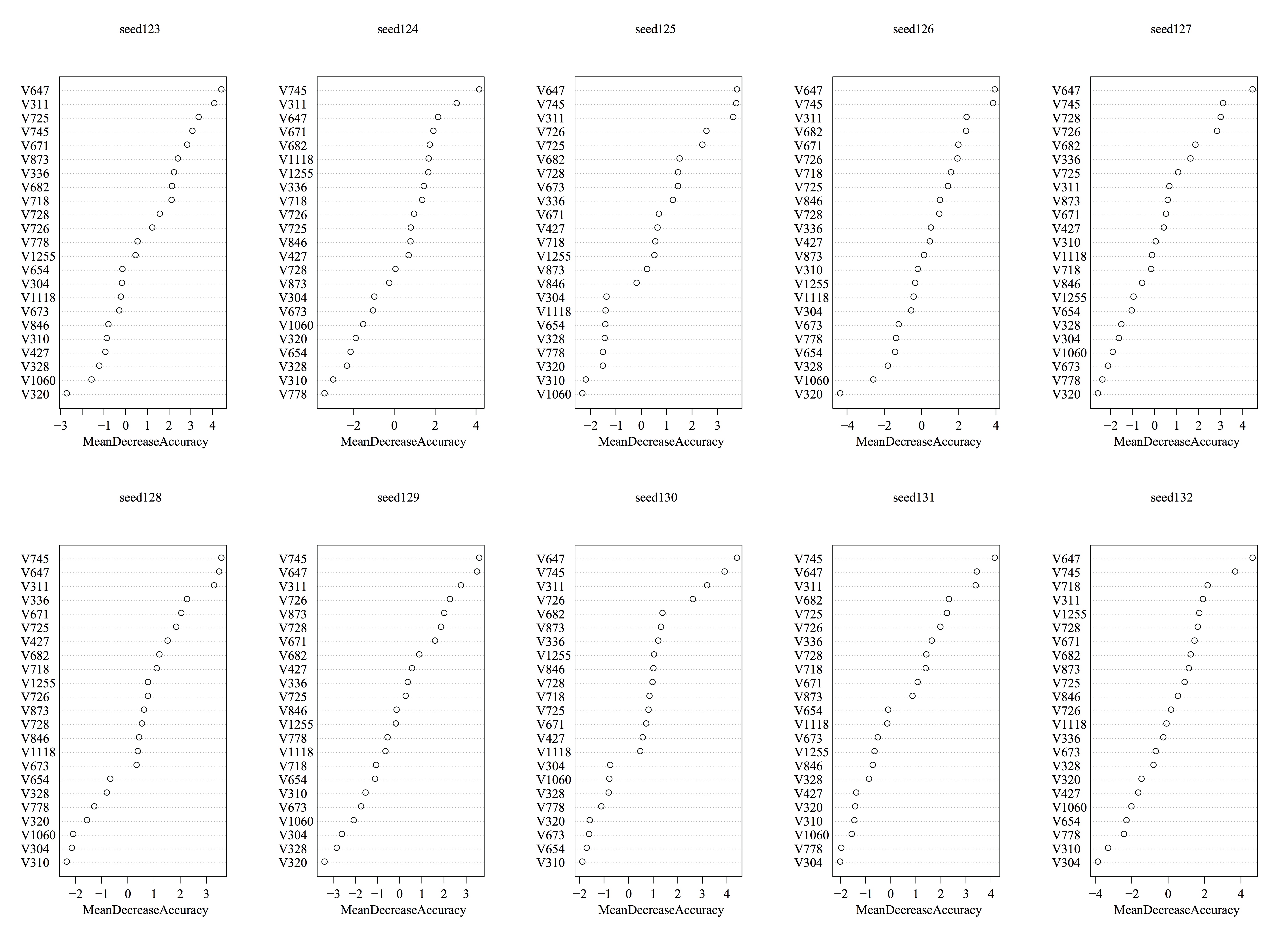

Supplement: Supplementary file 3 [file Image2.JPEG]
