# Supplementary material for: EasyMicroPlot: An Efficient and Convenient R Package in Microbiome Downstream Analysis and Visualization for Clinical Study
Source: Front Genet. 2022 Jan 4;12:803627. doi: 10.3389/fgene.2021.803627 (PMC8764268; doi:10.3389/fgene.2021.803627)
Supplement: Supplementary file 4 [file Table2.DOCX]

|  | **Control (N=464)** | **Cases (N=154)** | **Overall (N=618)** |
| --- | --- | --- | --- |
| **Aginomoto** |  |  |  |
| Mean (SD) | 18.3 (34.7) | 20.7 (33.2) | 18.9 (34.3) |
| Median [Min, Max] | 0 [0, 200] | 5.00 [0, 200] | 0 [0, 200] |
| Missing | 14 (3.0%) | 3 (1.9%) | 17 (2.8%) |
| **Animal_oil** |  |  |  |
| Mean (SD) | 23.6 (109) | 29.0 (98.0) | 24.9 (106) |
| Median [Min, Max] | 0 [0, 1250] | 0 [0, 571] | 0 [0, 1250] |
| Missing | 11 (2.4%) | 3 (1.9%) | 14 (2.3%) |
| **Plant_oil** |  |  |  |
| Mean (SD) | 1230 (824) | 1160 (735) | 1210 (802) |
| Median [Min, Max] | 1000 [0, 5330] | 1000 [0, 5000] | 1000 [0, 5330] |
| Missing | 18 (3.9%) | 5 (3.2%) | 23 (3.7%) |
| **Salt** |  |  |  |
| Mean (SD) | 116 (72.7) | 112 (63.1) | 115 (70.4) |
| Median [Min, Max] | 100 [0, 400] | 100 [0, 357] | 100 [0, 400] |
| Missing | 19 (4.1%) | 9 (5.8%) | 28 (4.5%) |
| **Sauce** |  |  |  |
| Mean (SD) | 24.8 (62.5) | 27.2 (68.9) | 25.4 (64.1) |
| Median [Min, Max] | 0 [0, 500] | 0 [0, 500] | 0 [0, 500] |
| Missing | 8 (1.7%) | 3 (1.9%) | 11 (1.8%) |
| **Soy_sauce** |  |  |  |
| Mean (SD) | 217 (467) | 217 (186) | 217 (415) |
| Median [Min, Max] | 145 [0, 6670] | 167 [0, 1000] | 150 [0, 6670] |
| Missing | 15 (3.2%) | 6 (3.9%) | 21 (3.4%) |
| **Sugar** |  |  |  |
| Mean (SD) | 43.6 (82.2) | 32.4 (53.3) | 40.8 (76.1) |
| Median [Min, Max] | 12.0 [0, 750] | 8.00 [0, 286] | 10.5 [0, 750] |
| Missing | 16 (3.4%) | 4 (2.6%) | 20 (3.2%) |
| **Fruit_juice** |  |  |  |
| Mean (SD) | 7040 (19800) | 6990 (19000) | 7030 (19600) |
| Median [Min, Max] | 0 [0, 183000] | 0 [0, 128000] | 0 [0, 183000] |
| **Fruits** |  |  |  |
| Mean (SD) | 69500 (66100) | 74800 (63700) | 70800 (65500) |
| Median [Min, Max] | 54800 [0, 548000] | 73000 [0, 383000] | 54800 [0, 548000] |
| **Grains** |  |  |  |
| Mean (SD) | 82800 (56700) | 84200 (51400) | 83200 (55400) |
| Median [Min, Max] | 73000 [0, 377000] | 73000 [2190, 329000] | 73000 [0, 377000] |
| **Red_wine** |  |  |  |
| Mean (SD) | 2180 (12700) | 2730 (8510) | 2320 (11800) |
| Median [Min, Max] | 0 [0, 234000] | 0 [0, 83200] | 0 [0, 234000] |
| **Rice_wine** |  |  |  |
| Mean (SD) | 342 (2770) | 125 (876) | 288 (2440) |
| Median [Min, Max] | 0 [0, 54800] | 0 [0, 7800] | 0 [0, 54800] |
| **H_alcohol** |  |  |  |
| Mean (SD) | 758 (3630) | 2150 (7160) | 1110 (4790) |
| Median [Min, Max] | 0 [0, 39000] | 0 [0, 54800] | 0 [0, 54800] |
| **L_alcohol** |  |  |  |
| Mean (SD) | 220 (1430) | 228 (1180) | 222 (1370) |
| Median [Min, Max] | 0 [0, 18300] | 0 [0, 10400] | 0 [0, 18300] |
| **Livestock** |  |  |  |
| Mean (SD) | 33200 (31400) | 34800 (30300) | 33600 (31100) |
| Median [Min, Max] | 23500 [0, 183000] | 26600 [0, 183000] | 23900 [0, 183000] |
| **Vegetables** |  |  |  |
| Mean (SD) | 127000 (82500) | 130000 (95800) | 127000 (85900) |
| Median [Min, Max] | 110000 [0, 548000] | 110000 [14600, 821000] | 110000 [0, 821000] |
| **Diarrhea** |  |  |  |
| n | 434 (93.5%) | 139 (90.3%) | 573 (92.7%) |
| y | 26 (5.6%) | 15 (9.7%) | 41 (6.6%) |
| Missing | 4 (0.9%) | 0 (0%) | 4 (0.6%) |
| **Astriction** |  |  |  |
| n | 420 (90.5%) | 141 (91.6%) | 561 (90.8%) |
| y | 40 (8.6%) | 13 (8.4%) | 53 (8.6%) |
| Missing | 4 (0.9%) | 0 (0%) | 4 (0.6%) |
| **Antibiotics** |  |  |  |
| n | 414 (89.2%) | 146 (94.8%) | 560 (90.6%) |
| y | 45 (9.7%) | 7 (4.5%) | 52 (8.4%) |
| Missing | 5 (1.1%) | 1 (0.6%) | 6 (1.0%) |
| **Synbiotics** |  |  |  |
| n | 394 (84.9%) | 137 (89.0%) | 531 (85.9%) |
| y | 64 (13.8%) | 16 (10.4%) | 80 (12.9%) |
| Missing | 6 (1.3%) | 1 (0.6%) | 7 (1.1%) |
